# Prognostic Impact of Epidermal Growth Factor Receptor Overexpression in Patients with Cervical Cancer: A Meta-Analysis

**DOI:** 10.1371/journal.pone.0158787

**Published:** 2016-07-20

**Authors:** Wei-Jie Tian, Miao-Ling Huang, Qing-Feng Qin, Qing Chen, Kun Fang, Ping-Ling Wang

**Affiliations:** 1 Department of Obstetrics and Gynecology, Guizhou Provincial People’s Hospital, Guiyang, Guizhou, P. R. China; 2 Department of Obstetrics and Gynecology, Sun Yat-sen Memorial Hospital, Sun Yat-sen University, Guangzhou, Guangdong, P. R. China; University of South Alabama Mitchell Cancer Institute, UNITED STATES

## Abstract

Clinical trials have provided conflicting results regarding whether epidermal growth factor receptor (EGFR) overexpression predicts poor survival in cervical cancer patients. In this study, we perform a meta-analysis of the association between EGFR expression and survival in cervical cancer patients. We searched clinical studies in the Medline, PubMed, Embase, and Web of Science databases. A total of 22 studies with 2,505 patients were included, and pooled hazard ratios (HRs) with 95% confidence intervals (CIs) were calculated for each study. Heterogeneity was assessed using Higgins *I*^*2*^ to select a Mantel-Haenszel fixed effects model (*I*^*2*^ ≤50%) or a DerSimonian-Laird random effects model (*I*^*2*^ ≥50%). High EGFR levels predicted poor overall survival (OS) (HR: 1.40, 95% CI: 1.10–1.78) and disease-free survival (DFS) (HR: 1.84, 95% CI: 1.51–2.24). Stratified analyses showed that EGFR overexpression was significantly related to poor DFS in patients treated with chemoradiation or surgery. Moreover, the pooled odds ratios (ORs) revealed associations between EGFR expression and clinicopathological features, such as lymph node metastasis (OR: 1.72, 95% CI: 1.23–2.40) and tumor size ≥4 cm (OR: 1.64, 95% CI: 1.20–2.23). This meta-analysis demonstrates that EGFR overexpression is closely associated with reduced survival in patients with cervical cancer. These results may facilitate the individualized management of clinical decisions for anti-EGFR therapies in cervical cancer patients.

## Introduction

Cervical cancer is the third most frequently diagnosed malignancy and represents the fourth leading cause of cancer-related death in females worldwide [1]. With the introduction of screening programs, the incidence of and mortality associated with cervical cancer in developed areas have dramatically declined in recent decades [2]. The standard treatment for locally advanced cervical cancer consists of concurrent platinum-based chemoradiation, which results in a 5-year survival rate of only 66% [3]. Tremendous efforts are still needed to improve the overall survival rate in patients with advanced-stage cervical cancer.

Epidermal growth factor receptor (EGFR) is a 170-kDa transmembrane glycoprotein receptor dimerizes to activate a tyrosine kinase domain that modulates multiple functions, including cell differentiation, growth, gene expression, and development [4]. Because EGFR is known to play a role in epithelial tumor biology, various EGFR-targeted cancer therapies are currently being developed. EGFR inhibitors have demonstrated efficacy in some clinical trials involving patients with colon, lung, head, and neck cancers [5–7]. However, the value of using EGFR inhibition to treat cervical cancer remains unknown. Several small-scale clinical trials of EGFR inhibitors have been completed in cervical cancer patients, but the effects of these drugs are not yet well established [8–12]. Numerous clinical trials have demonstrated that only a subset of patients respond to EGFR inhibitors. However, a practical predictor of a response to these drugs has not been identified for cervical cancer.

The overexpression of EGFR is thought to be negatively associated with survival in cervical cancer patients, and the relationship between EGFR overexpression and altered survival in patients with cervical cancer has therefore been studied for many years [13]. However, inconclusive results have been reported by different laboratories. A meta-analysis is needed to comprehensively evaluate the prognostic value of EGFR in this type of malignancy. Therefore, we performed a systematic meta-analysis to quantify the effects of EGFR overexpression on survival in patients with cervical cancer.

## Materials and Methods

### Search strategy

The Medline, PubMed, Embase, and Web of Science databases (through March 2014) were searched to identify articles that examined EGFR expression status and survival in patients with cervical cancer using combinations of the following terms: EGFR (or epidermal growth factor receptor, Her family, Her-1, Erb B family, or Erb B1), outcome (or surviv*, prognos*, or predict*), and cervical cancer (or cervical carcinoma, cervical neoplasm, or cervical tumor). The references of all resulting publications and reviews were manually searched to identify missing relevant publications. All studies were carefully evaluated to identify duplicate data.

### Selection criteria

The following criteria for study eligibility were set before articles were collected: (1) EGFR was evaluated in primary cervical cancer tissues using immunohistochemistry (IHC) or by quantifying EGFR protein levels; (2) a hazard ratio (HR) and its confidence interval (CI) from a survival analysis were reported; (3) the median follow up time exceeded 2 years; (4) the investigated endpoints were overall survival (OS) and disease-free survival (DFS); and (5) when a single study was reported on multiple occasions, the latest or most informative article was selected.

### Data extraction

Two authors (W-J Tian and M-L Huang) independently extracted information using predefined data abstraction forms. Further information from each study is shown in S1 Table. If a study reported the results of both univariate and multivariate analyses, the latter was selected because multivariate analyses consider confounding factors, which makes them more precise.

### Quality assessment of primary studies

The quality of each study was independently assessed by two investigators (W-J Tian and M-L Huang) using the criteria developed by McShane et al. [14] and Hayes et al. [15] (S2 Table). The following eight items were assessed and scored on a scale ranging from 0 to 8: 1) the study reported inclusion and exclusion criteria, 2) the study design was prospective or retrospective, 3) the patient and tumor characteristics were sufficiently described, 4) the method or assay used to measure biomarker expression was sufficiently described, 5) a description of the study endpoint was provided, 6) the duration of the follow-up period in the study was provided, 7) the study reported the number of patients who dropped out during the follow-up period or for whom data was not available for statistical analysis, and 8) staining was evaluated by more than one observer. Studies with a total score of eight were considered to have used the highest quality methodology, whereas a score of zero was considered to indicate a study with the lowest quality methodology.

All disagreements were resolved by discussion with the third author (P-L Wang).

### Statistical analysis

All statistical methods used in this study were performed using Stata statistical software (version 12.0; Stata Corporation, College Station, TX, USA). The HRs and associated 95% Cis that were obtained from original articles were directly extracted or estimated from available data using methods previously reported by Tierney et al. [16]. For the pooled analysis of the relationship between EGFR expression and clinicopathological parameters, odds ratios (ORs) and their 95% CIs were combined to determine effective values. The point estimate of the HR or OR was considered statistically significant at a level of *P*<0.05 if the 95% CI did not include the value “1”.

DerSimonian-Laird random effects analysis [17] and the Mantel-Haenszel fixed effects method [18] were used to calculate the pooled HRs/ORs. The heterogeneity assumption was tested using Cochran’s *Q* test and simultaneously quantified using the Higgins *I*^*2*^ statistic [19]. A value of *P*>0.1 was regarded as indicating a lack of heterogeneity, while *I*^*2*^>50% indicated substantial heterogeneity. Sensitivity analyses were conducted to evaluate the stability of the results. An evaluation of potential publication bias was performed using a funnel plot. The asymmetry of the funnel plot was also assessed using Egger’s test [20].

## Results

### Study characteristics

**Fig 1:**
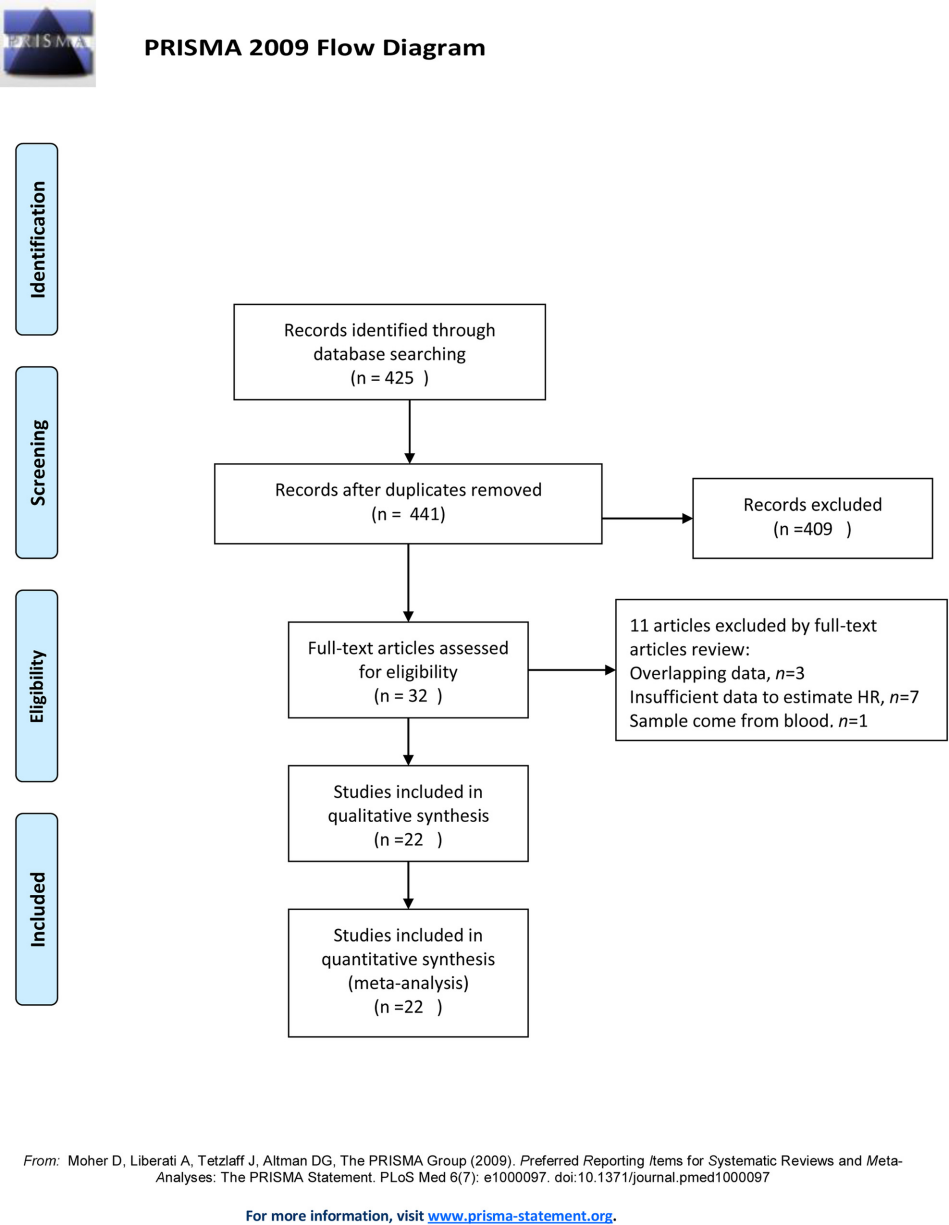
Study Selection Flowchart.

A total of 441 records were identified from a search of the primary literature that involved screening the title and abstract for the research results (Fig 1). Based on the inclusion criteria listed in a previous sections, 32 papers were eligible for further assessment. Overlap between patients was discovered among three studies [21–23]. The study [21] with the most recent data was selected for inclusion in the overall analysis. Seven studies were excluded because insufficient data were provided to estimate HRs (for an overview, see S3 Table). One study [24] that explored the relationship between circulating EGFR levels and cervical cancer was not excluded because it reduced the source of heterogeneity. In addition, two studies [25,26] involving data obtained from the same medical center had different inclusion criteria for the observed populations and treatments, and both of these studies were therefore included. One study [27] examined squamous cell carcinoma and adenocarcinoma separately, and the data from this study were therefore considered to represent two studies. Therefore, 22 studies [21,25–45] involving 2,505 patients were included in the final meta-analysis. The main characteristics of the 22 eligible studies are summarized in S1 Table. Among the studies included in this meta-analysis, 11 (52.4%) reported a significant association between EGFR overexpression and survival, including 10 (47.6%) that concluded that EGFR overexpression was associated with shorter survival and one (4.8%) that reported that EGFR expression was associated with longer survival. The remaining 11 reports (47.6%) yielded insignificant results.

The rate of positivity for EGFR overexpression in individual studies ranged from 18.0% to 87.3%. When subdivided according to the histological type of tissue, EGFR positivity was found to be 47.9% in squamous cell carcinomas and 38.2% in adenocarcinomas. The data were insufficient to analyze EGFR overexpression positivity in other histotypes. Overall, OS was extracted from 19 studies [21,26–28,30–39,41,42,44,45]. Among these studies, the predominant treatments included surgery in six studies and chemoradiation in nine studies. The remaining studies used mixed treatments or unknown methods. DFS was obtained from 11 studies [21,25,26,29,32,35,37,40,42,43,45]. Among these studies, the predominant treatment was surgery in four studies and chemoradiation in six studies.

### Pooled analysis

#### EGFR expression and OS in cervical cancer patients

**Fig 2:**
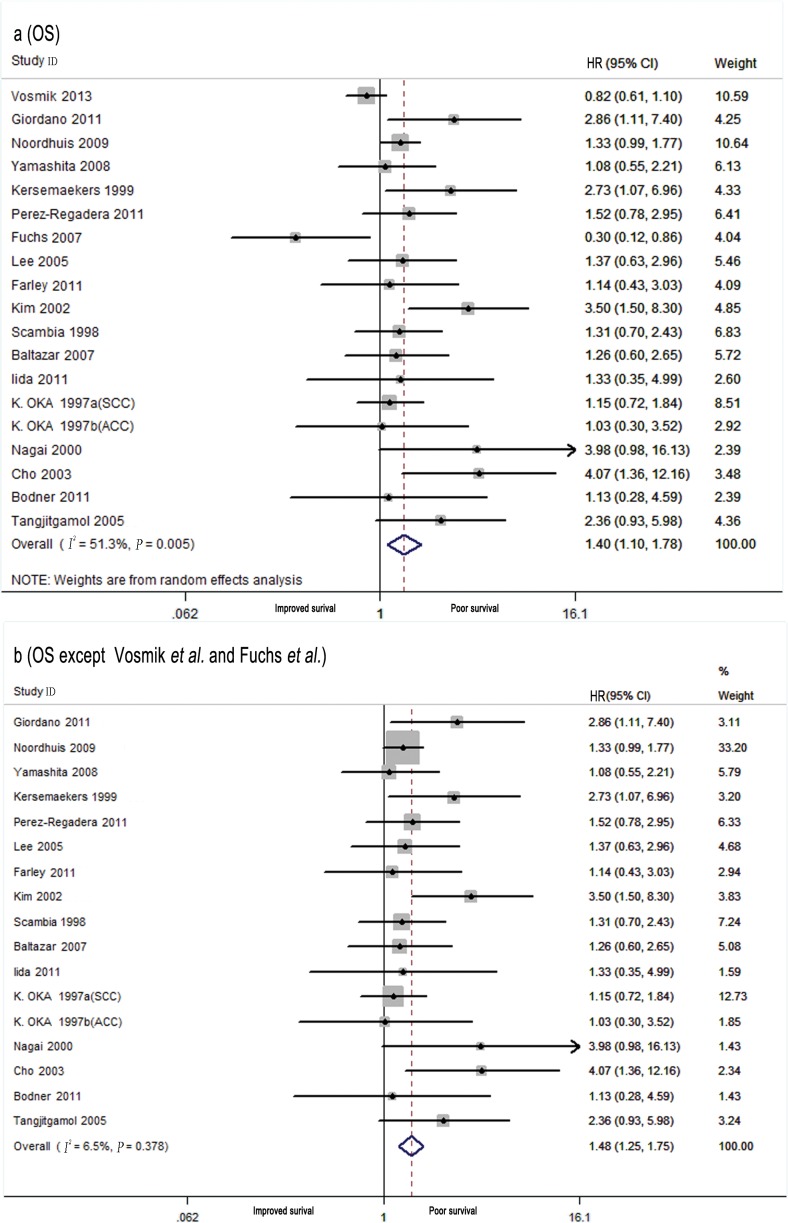
Meta-analysis of the Predictive and Prognostic Value of EGFR Expression for Determining Overall Survival (OS). Each study is shown as the point estimate of its hazard ratio (HR) (the size of the square is proportional to the weight of each study) and the 95% confidence interval (CI) for the HR (horizontal bars). a, OS of all studies; b, OS of all studies except Vosmik et al. [28] and Fuchs et al. [39].

Table 1 demonstrates the main result of the meta-analysis results. Overall, in cervical patients, high EGFR levels in the primary tumor were significantly associated with poor OS in the random effects model (combined HR: 1.40, 95% CI: 1.10–1.78; Fig 2A) despite the presence of heterogeneity between the studies (*I*^*2*^ = 51.3%, *P*_*h*_ = 0.005). To identify the sources of this heterogeneity, subgroup analyses and meta-regressions were performed by treatment, histological type, quality rating score, number of patients, publication year, study design, and study location. Heterogeneity was found to be associated with study quality, the number of patients, the publication year, and the study design. When data related to these four characteristics were restricted (quality rating score ≥6, number of patients ≥100, publication year <2007, and prospective studies), the heterogeneity was substantially decreased and the pooled results remained practically unchanged (Table 1). Further meta-regression analyses showed that the publication year might account for part of the observed inter-study heterogeneity (*P* = 0.047). In studies performed after 2007, heterogeneity was mainly due to the results of studies by Vosmik et al. [28] and Fuchs et al. [39]. When these two studies were excluded from the meta-analysis, there was no heterogeneity (*I*^*2*^ = 6.5%, *P*_*h*_ = 0.378), and the pooled results remained practically unchanged (HR: 1.48, 95% CI: 1.25–1.75; Fig 2B) (Table 1). The subgroup analysis indicated the presence of an association between EGFR overexpression and OS in studies with a quality rating score ≥6 (HR: 1.42, 95% CI: 1.41–1.76), studies with ≥100 patients (HR: 1.40, 95% CI: 1.34–1.73), prospectively designed studies (HR: 1.49, 95% CI: 1.18–1.88), studies published before 2007 (HR: 1.69; 95% CI: 1.30–2.20), and studies performed in Asia (HR: 1.76, 95% CI: 1.09–2.82) (Table 1).

**Table 1:** Main Results of the Pooled Analysis.

Analysis	N	References	Random-effects model		Fixed-effects model		Meta-regression P-value	Heterogeneity	
			Pooled HR	95% CI of HR	Pooled HR	95% CI of HR		*I*^*2*^ test (%)	*P*-value
**Overall survival (OS)**	19	21, 26–28, 30–39, 41, 42, 44, 45	1.40	1.10–1.78	1.24	1.08–1.44		51.30%	0.005
**OS (except Vosmik, M.; Fuchs, I.)**	17	21, 26–27, 30–38, 41, 42, 44, 45	1.51	1.26–1.81	1.48	1.25–1.75		6.50%	0.378
**Subgroup 1: Treatment**							0.580		
Chemoradiation	9	21, 26–28, 32, 37, 38, 41	1.19	0.94–1.51	1.14	0.96–1.34		33.60%	0.149
Surgery	6	30, 31, 34, 35, 39, 45	1.53	0.67–3.48	1.51	0.96–2.37		68.10%	0.008
**Subgroup 2:Quality rating score**							0.632		
Score ≥6	6	26, 31, 37, 32, 33, 36	1.42	1.14–1.76	1.42	1.41–1.76		0.00%	0.53
Score ≤5	13	21, 27, 28, 30, 34, 35, 38, 39, 41, 42, 44, 45	1.37	0.95–1.97	1.12	0.93–1.36		60.50%	0.002
**Subgroup 3: Histological type**									
SCC	8	27, 28, 30, 31, 32, 39, 41	1.17	0.73–1.87	0.99	0.80–1.23		68.50%	0.004
Other type	—	—	—	—	—	—		—	—
**Subgroup 4: No. of patients**							0.590		
≥100	7	26, 27, 31, 35, 37, 44	1.41	1.34–1.74	1.40	1.34–1.73		1.30%	0.48
<100	12	21, 28, 30, 32, 33, 34, 36, 38, 39, 41, 42, 45	1.35	0.94–1.96	1.12	0.92–1.36		59.40%	0.003
**Subgroup 5: Publication year**							0.047		
≥2007	10	26, 28, 30, 31, 32, 37, 38, 39, 44, 45	1.12	0.84–1.50	1.09	0.92–1.30		47.50%	0.046
<2007	9	21, 27, 33–36, 41, 42	1.86	1.31–2.63	1.69	1.30–2.20		35.10%	0.137
**Subgroup 6: Study design**							0.295		
prospective	5	26, 34, 36, 37, 42	1.66	1.52–2.38	1.49	1.18–1.88		38.20%	0.166
retrospective	14	21, 27, 28, 30–33, 35, 38, 39, 41, 44, 45	1.29	0.95–1.75	1.11	0.92–1.33		51.20%	0.014
**Subgroup 7: Study location**							0.132		
Europe	8	26, 28, 31, 35–37, 45, 39	1.23	0.84–1.73	1.13	0.95–1.35		66.30%	0.004
Asia	7	27, 30, 32, 34, 41, 42	1.76	1.09–2.82	1.54	1.13–2.10		46.80%	0.08
America	4	21, 32, 33, 44	1.44	0.95–2.19	1.44	0.95–2.19		0.00%	0.696
**Disease-free survival (DFS)**	11	21, 25, 26, 29, 32, 35, 37, 40, 42, 43, 45	1.91	1.50–2.44	1.84	1.51–2.24		23.90%	0.216
**Subgroup 1: Treatment**									
Chemoradiation	6	21, 26, 29, 32, 37, 40	1.70	1.30–2.23	1.69	1.32–2.15		10.80%	0.346
Surgery	4	25, 35, 43, 45	2.15	1.27–2.63	2.02	1.37–2.96		40.20%	0.17
**Subgroup 2: Quality rating score**											
Score ≥6	6	25. 26, 29, 32, 37, 40	1.67	1.29–2.15	1.65	1.31–2.07		13.70%	0.327
Score ≤5	5	21, 35, 42, 43, 45	2.55	1.72–3.77	2.55	1.72–3.77		0.00%	0.435
**Subgroup 3: Histological type**									
SCC	4	29, 32, 40, 43	2.22	1.30–3.49	2.06	1.43–3.01		37.60%	0.186
Other type	—	—	—	—	—	—		—	—
**Subgroup 4: No. of patients**									
≥100	6	25, 26, 29, 35, 37, 43	1.90	1.43–2.52	1.82	1.45–2.28		25.30%	0.244
<100	5	21, 32, 40, 42, 45	1.93	1.16–3.20	1.91	1.29–2.84		37.50%	0.171
**Subgroup 5: Publication year**									
≥2007	6	25, 26, 29, 32, 37, 45	1.59	1.26–2.00	1.59	1.26–2.00		0.00%	0.594
<2007	5	21, 35, 40, 42, 43	2.70	1.85–3.94	2.70	1.85–3.94		0.00%	0.411
**Subgroup 6: Study design**									
prospective	5	25, 26, 29, 37, 42	1.78	1.35–2.35	1.74	1.37–2.21		18.30%	0.298
retrospective	6	21, 32, 35, 40, 43, 45	2.10	1.34–3.28	2.09	1.46–2.98		33.70%	0.183
**Subgroup 7: Study location**									
Europe	7	25, 26, 29, 35, 37, 43, 45	1.87	1.46–2.41	1.83	1.46–2.29		11.80%	0.34
Asia	3	32, 40, 42	2.20	1.07–4.48	2.12	1.34–3.34		58.50%	0.09
America	1	21	—	—	—	—		—	—
**Clinicopathological parameters**			Pooled OR	95% CI of OR	Pooled OR	95% CI of OR			
Age (old vs. young)	4	26, 32, 36, 45	1.00	0.99–1.02	1.00	0.99–1.02		0.00%	0.47
Lymph node metastasis (Yes vs. No)	7	26, 29, 32, 35, 36, 43, 45	1.72	1.23–2.40	1.72	1.23–2.40		0.00%	0.862
Tumor grade (grade 3 vs. grade 1 and grade 2)	7	26, 35, 36, 40, 43, 45, 46	0.74	0.51–1.05	0.74	0.55–1		16.10%	0.307
Tumor size (size ≥4 cm vs. size <4 cm)	7	26, 32, 35, 36, 40, 43, 45	1.64	1.20–2.23	1.64	1.20–2.23		0.00%	0.935
FIGO stage (stage III/IV vs. stage IB/II)	6	26, 29, 35, 36, 45, 46	1.37	0.74–2.53	1.34	0.98–1.84		67.10%	0.01

#### EGFR expression and DFS in cervical cancer

**Fig 3:**
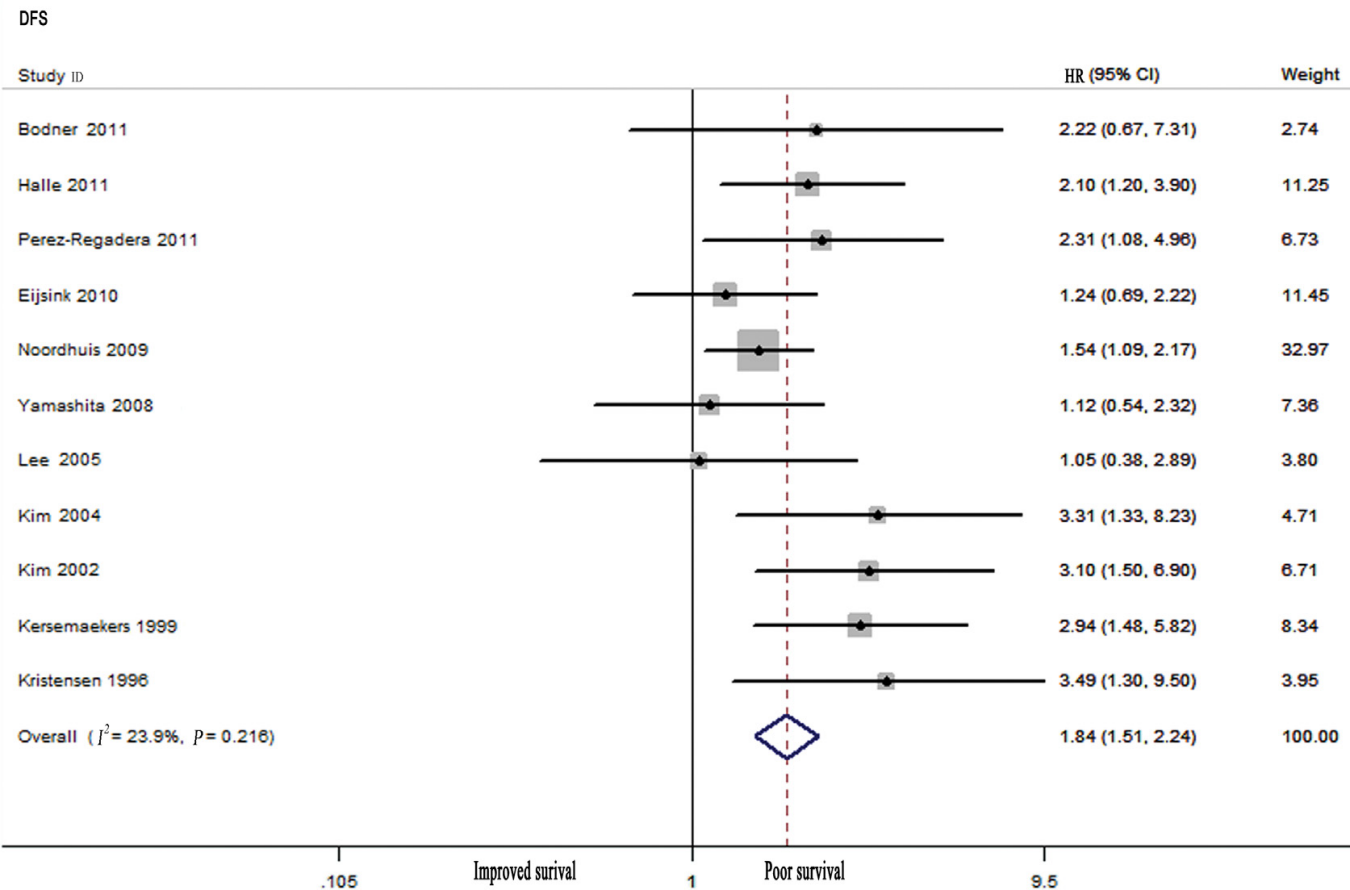
Meta-analysis of the Predictive and Prognostic Value of EGFR Expression for Predicting Disease-free Survival (DFS).

A meta-analysis of 11 studies revealed that high EGFR levels were associated with poor DFS (HR: 1.84, 95% CI: 1.51–2.24; Fig 3), and no significant heterogeneity was observed (*I*^*2*^ = 23.9%, *P*_*h*_ = 0.216). Subgroup analyses demonstrated that EGFR overexpression was related to poor DFS in cervical cancer patients who were treated with chemoradiation (HR: 1.69, 95% CI: 1.32–2.15) and surgery (HR: 2.02, 95% CI: 1.37–2.96). Other subgroup analyses performed by quality rating score, histological type, number of patients, publication year, study design, and study location also revealed associations between high EGFR levels and poor DFS in cervical cancer patients. There was no obvious heterogeneity in these data, except in studies that were conducted in Asia (Table 1).

#### EGFR expression and clinicopathological characteristics

Seven studies [26,29,32,35,36,43,45] assessed the relationship between EGFR overexpression and lymph node status. The pooled data showed that patients with lymph node metastasis exhibited significantly higher levels of EGFR expression than were observed in patients without metastasis, with a combined OR of 1.72 (95% CI: 1.23–2.40) and without heterogeneity (*I*^*2*^ = 0.00%, *P*_*h*_ = 0.862). After combining the seven studies [26,32,35,36,40,43,45] that examined tumor size, we also observed a statistically significant effect of high EGFR levels on tumor sizes ≥4 cm, with a combined OR of 1.64 (95% CI: 1.20–2.23) and no significant heterogeneity (*I*^*2*^ = 0.00%, *P*_*h*_ = 0.935). Furthermore, four studies [26,32,36,45] reported data on age, seven studies [26,35,36,40,43,45,46] reported the tumor grade, and six studies [26,29,35,36,45,46] reported the FIGO stage. The pooled results indicated that there was no significant association between high EGFR expression levels and age (OR: 1, 95% CI: 0.99–1.02), tumor grade (OR: 0.74, 95% CI: 0.51–1.05), or FIGO stage (OR: 1.37, 95% CI: 0.74–2.53) (Table 1).

#### Sensitivity analyses and publication bias

The sensitivity analysis indicated that omitting any single study did not significantly affect the combined HR, supporting the robustness of the HR estimates.

We constructed Begg’s funnel plots and conducted Egger’s tests to evaluate publication bias. The shape of the funnel plots revealed no significant asymmetry (figures not shown). Egger’s tests also demonstrated no any evidence of obvious publication bias among the studies included in the overall analyses of OS (*P* = 0.068) and DFS (*P* = 0.205).

## Discussion

EGFR monoclonal antibodies have been applied as efficacious adjuvant treatments in solid tumors, such as colon and lung cancer. However, data related to the effect of EGFR inhibitors on cervical cancer remain inconclusive. Correctly evaluating the association between EGFR expression and survival in cervical cancer patients is essential to understanding the mechanisms of action underlying anti-EGFR therapies. The results that have been reported in other studies have been controversial. Therefore, we performed this meta-analysis to determine whether EGFR overexpression could predict prognoses in cervical cancer patients.

To our knowledge, this is the first meta-analysis to study the association between EGFR expression and OS, DFS, and clinicopathological characteristics in cervical cancer patients. Our combined analysis of 23 published studies that included 2,505 patients with cervical cancer yielded summary statistics demonstrating that in cervical cancer patients, high EGFR levels are associated with lower OS and DFS (Table 1).

In the studies included in this meta-analysis, significant heterogeneity was observed for OS. To explore the sources of this heterogeneity, we performed a sensitivity analysis, subgroup analysis, and meta-regression and found that the heterogeneity was mainly because of the studies by Vosmik et al. [28] and Fuchs et al. [39]. Further analysis revealed that both studies reported a favorable trend for cervical cancer patients with EGFR overexpression. This interesting result may have occurred because both studies simultaneously explored EGFR and other HER family members. Fuchs et al. [39] reported that the prognostic impact of HER1 (EGFR) or HER2 was often dependent on the correlation with HER3 or HER4. Therefore, further studies of the prognostic effects of HER family combinations are needed. Some other sources of heterogeneity might also exist in this meta-analysis. This include the method used to detect EGFR expression, the IHC scoring system, the cut-off values used for high EGFR expression, and differences in patients (e.g., differences in their ages, clinical stages, and physical conditions). Because of data limitations, we were unable to comprehensively analyze these aspects. The sources of heterogeneity that were identified indicate that using a consistent, standard study design and a larger sample size are necessary to obtain reliable results. Interestingly, no obvious evidence of data heterogeneity was found for DFS except for the studies conducted in Asia. Many of the included DFS studies met the criteria related to higher quality rating scores and larger numbers of patients. We also found that high EGFR levels were significantly correlated with poor OS and DFS in patients in studies performed in Asia (OS: HR: 1.76, 95% CI: 1.09–2.82; DFS: HR: 2.2, 95% CI: 1.07–4.48) but not in studies performed in other locations. This may be because the characteristics of cervical cancer might vary between geographical regions as a result of a variety of environmental factors and race-related genetic effects. However, significant heterogeneity was observed in both subgroup analyses. Thus, it is essential that more should be performed to determine whether EGFR overexpression is a prognostic factor for cervical cancer patients in Asia and other regions.

It has long been known that squamous cell carcinoma in cervical tumors differs in important ways from other histological subtypes [4]. In this study, we assessed the prognostic value of EGFR expression in squamous cell carcinoma. A significant association was observed between high EGFR levels and poor DFS in squamous cell cervical cancer patients (HR: 2.06, 95% CI: 1.43–3.01). However, the result for OS was not of prognostic value (HR: 1.17, 95% CI: 0.73–1.87), and significant heterogeneity was detected. Thus, these results should be interpreted with caution, and further studies are needed. Moreover, the number of studies of other histological subtypes is limited, and we were unable to conduct a subgroup analysis. Thus, further research regarding adenocarcinoma or adenosquamous cell carcinoma is encouraged.

EGFR inhibitors mainly target activating *EGFR* mutations in non-small-cell lung cancer (NSCLC), and EGFR expression and EGFR mutations have been investigated as potential predictors of responsiveness to EGFR tyrosine kinase inhibitors in NSCLC [47]. Sonobe et al. [48] reported that EGFR gene mutations were significantly associated with higher EGFR expression in patients with NSCLC. It is therefore reasonable to assume that there is a relationship between EGFR gene mutations and EGFR expression and that EGFR gene mutations may also be useful for predicting responses to EGFR inhibitors in patients with cervical cancer. However, because of EGFR mutations are very rare in cervical cancer [49] and data limitations, we were unable to analyze these hypotheses. They may be important areas for future work.

Some limitations were present in this meta-analysis. First, it has been shown that trials that provide negative results are usually only briefly reported or reported in non-English languages. This phenomenon was observed in this meta-analysis, although Begg’s funnel plots and Egger’s tests did not show any clear evidence of publication bias. Second, this meta-analysis was performed to analyze the results of observational trials, and more confounding factors are present in these types of trials than in randomized controlled trials. However, because of data limitations, we were unable to assess these factors in this study. Third, HRs that are extrapolated from survival curves or other relevant data might be less reliable than HRs that are obtained directly from articles. Hence, because of the limitations of this meta-analysis, the value of EGFR expression as a prognostic indicator in cervical cancer should be confirmed in future well-designed prospective clinical trials.

Despite its limitations, the results of the current stratified analysis, sensitivity analysis, and random and fixed effects models demonstrate the robustness of this meta-analysis.

## Conclusions

This meta-analysis reveals that EGFR overexpression might be a predictive biomarker of reduced survival in patients with cervical cancer. This finding could potentially affect clinical decision-making and ultimately result in more effective targeted therapies for cervical cancer patients.

## Supporting Information

S1 Table Main Characteristics of all Studies Included in the Meta-analysis. (DOC)

S2 Table Quality Rating Score of the Included Studies. (DOC)

S3 Table Overview of Studies that were Excluded because of Insufficient Data. (DOC)

S4 Table PRISMA Checklist. (DOC)
