# Supplementary material for: Prognostic Impact of Epidermal Growth Factor Receptor Overexpression in Patients with Cervical Cancer: A Meta-Analysis
Source: PLoS One. 2016 Jul 20;11(7):e0158787. doi: 10.1371/journal.pone.0158787 (PMC4954718; doi:10.1371/journal.pone.0158787)
Supplement: S1 Table — (DOC) [file pone.0158787.s001.doc]

Supplementary table 1. Main characteristics of all studies included in the meta-analysis

| Study | country | Treatment (predominant) | No. in study  (of deaths  /recurrences) | Inclusion period | sources of specimen | Age in years | FIGO stage | Histological type | Assay (p/m) | Method to determine "high"EGFR cut-off level | Number of patients with "high" EGFR | Follow-up in months* | Quality rating | Survival analysis | HR |
| --- | --- | --- | --- | --- | --- | --- | --- | --- | --- | --- | --- | --- | --- | --- | --- |
| Milan Vosmik 2013 | Czech Republic | RT, CRT (CR) | 70(36) | Aug.,1998 to Aug.,2008 | paraffin blocks | Median 50; (range 28-81) | IB:1; IIA:2 IIB:25; IIIB:40 IVB:4; | SCC:72 | IHC(m) | grade≥2 of cells staining moderate or strong intensity | 54 | median 57 (3–153 ) | 5 | OS | R |
| Halle, C. 2011 | Norway | CRT (CR) | 178（50） | 1999 to 2006 | paraffin blocks | Median 56; (range 25-85) | IB:9; II:106 III:53;IVA:10 | SCC:178 | IHC(m) | ≥10% of cells staining positive | 32 | median 49 (18–93 ) | 6 | DFS | R |
| Giovanna 2011 | Italy | S±CHT/ RT (S) | 102(23) | 1993 to 2009 | paraffin blocks | NA | NA | SCC:110 | IHC(m) | ≥10% of cells staining positive | 77 | range<12–168 | 7 | OS | E |
| Eijsink, J. J2010 | Netherlands | S±RT/ CHT (S) | 336(20) | Jan.,1980 to Dec., 2004 | paraffin blocks | Median 43; (range 17-86) | IB_1_:197; IB_2_:63 IIA:50 | SCC:200 AC:87 Other:23 | IHC(NA) | ≥10% of cells staining positive | NA | median 66 (3.72– 223.2) | 7 | DFS | R |
| Noordhuis2009 | Netherlands | CRT (CR) | 375(195) | Jan.,1980 to Dec.,2006 | paraffin blocks | Median 54; (range21-92) | IB_1_:42;IB_2_:27 IIA:51;  IIB:179;  IIIA:11;  IIIB:51 IVA:14 | SCC:311 AC:52 Other:12 | IHC(NA) | ≥10% of cells staining positive | NA | median 40.8 (1.2 – 219.6) | 7 | OS;DFS | R (M) |
| Yamashita, H2008 | Japan | CRT (CR) | 57(34) | 1998 to 2005 | paraffin blocks | Median 61; (range27–82) | II:18; III;29 IVA:10 | SCC:57 | IHC(NA) | IRS≥5 | 26 | median 30.9 (4.0–120.8) | 6 | OS;DFS | E |
| Kersemaekers, A. M1999 | Netherlands | S (S) | 136(33) | 1984 to 1995 | paraffin blocks | Median 41; (range23–76) | IA_1_+ IB_1_:80 IB_2_:22; II:28 | SCC:115 AC:10 ASCC:9 | IHC(m) | score≥4 | 73 | median 48 (1 – 133 ) | 5 | OS;DFS | E |
| Perez-Regadera, J.2011 | Spain | CRT (CR) | 112(43) | Dec.,1994 to Sep.,2004 | paraffin blocks | NA | IB–IIA:11; IIB:25;  IIIB:63 IVA:13 | SCC:99 AC:11 ASCC:2 | IHC(m) | ≥31%of cells staining positive | 80 | median 46 (7 – 127) | 6 | OS;DFS | E |
| Fuchs, I.2007 | Germany | S/S+RT/CHT (S) | 78(NA) | 1987 to 1994 | paraffin blocks | Median 50; (range24–89) | IA:1; IB:33 IIA:6; IIB:16 IIIA:4; IIIB:12 IVA:3; IVB:3 | SCC | IHC(m) | IRS ≥6 | 49 | median 60 (1 – 180 ) | 3 | OS | R (M) |
| Lee, C. M2005 | American | RT(CR) | 55(NA) | 1981 to 1996 | paraffin blocks | NA | IB:12; II:22 III:17; IVA:4 | SCC:48 AC:5 ASCC:2 | IHC(m) | ＞7380 intensity scale | 48 | median 24 | 4 | OS;DFS | R (M) |
| Farley, J2011 | American | CHT(CR) | 48（42） | 2004 to 2008 | paraffin blocks | Median 51; (range24–77) | NA | NA | IHC(m) | median values of percentage | 25 | followed until death | 3 | OS | E |
| Kim, Y. T.2002 | Korea | S,nCHT+CRT (M) | 73（13） | Jan.,1993 to Jun.,1995 | Frozen tissue | Mean 49.8; (range34–78) | I–II:41; III–IV:32 | SCC:64 Other:9 | EIA | 250 fmol/mg | 52 | median59 (9 – 78 ) | 5 | OS;DFS | R (M) |
| Kristensen, G. B1996 | Norway | S/S+RT/CHT (S) | 132(23) | Jan.,1987 to Dec.,1990 | paraffin blocks | NA | IB | SCC | IHC(p) | ≥10% of cells staining positive | 34 | Median 73.9 (51.9 – 106) | 5 | DFS | R (M) |
| Scambia, G.1998 | Italy | nCHT+S,CRT,RT(M) | 88(40) | NA | Frozen tissue | NA | I–II:45 III–IV:45 | SCC:83 ASCC:7 | EIA | 6 fmol/mg | 45 | median 41 (2 – 120 ) | 6 | OS | E |
| Baltazar, F.2007 | Portugal and Brazil | NA | 129(NA) | 1986 to 2000 | paraffin blocks | Median 49.4; (range24–88) | NA | SCC:50 AC:50 ASCC:30 | IHC(p) | ≥5% of cells staining positive | 61 | NA | 2 | OS | E |
| Klida2011 | Japan | S,S+CRT/RT (M) | 59(NA) | Jan.,1994 and Dec.,2007 | paraffin blocks | Median 60; (range 26-84) | I:26; II:11 III:17; IVB:5; | SCC:59 | IHC(NA) | staining moderate to strong | 22 | Median 45 (5–120 ). | 4 | OS | E |
| K. OKA, 1997a(SCC) | Japan | RT(CR) | 216,only 187(NA) SCC included | NA | paraffin blocks | Mean 61; (range25–83) | III | SCC:191 AC:11 ASCC:14 | IHC(NA) | ≥5% of cells staining positive | 65 | 60 | 5 | OS | E |
| K. OKA, 1997b(ACC) | Japan | RT(CR) | 25 | NA | paraffin blocks | Mean61 (range25–83) | III | AC:11 ASCC:14 | IHC(NA) | ≥5% of cells staining positive | 4(16%) | 60 | 5 | OS | E |
| Nagai, N2000 | Japan | S,RT,CHT. (M) | 50(NA) | May,1991 to Dec.,1993 | Frozen tissue | NA | 0:1; IB:22; IIA:3; IIB:16; IIIA:0; IIIB:9 IVA:1 | SCC:43 AC:9 ASCC:1 | EIA | 20 fmol/mg | 16 | median 76 | 5 | OS | E |
| Cho, N. H.2003 | Korea | CRT(CR) | 84(NA) | 1991 to 1996 | paraffin blocks | NA | IIB | SCC | IHC(m) | ＞0% of nuclear staining positive | 47 | minimum follow-up of 60 | 5 | OS | E |
| Kim, G. E.2004 | Korea | CRT(CR) | 68(NA) | 1992 to 1996 | paraffin blocks | Mean 54; (range29–75) | IIB | SCC | IHC(m) | ≥10% of cells staining positive | 49 | median 66 (8 –108 ) | 6 | DFS | E |
| Bodner, K2011 | Austria | S+CRT,CRT (S) | 39(8) | 1994 to 2006 | paraffin blocks | Median 51; (range24-60) | I:17; II:15 III:6;IV:1 | AC | IHC(m) | ＞0% of cells staining positive | 17 | median 24 (2 – 123 ) | 4 | OS;DFS | E |
| Tangjitgamol, S2005^47^ | American | RT, CRT, S, S+ CRT (M) | 24(20) | Jan.,1975 to Dec.,1998 | paraffin blocks | mean age 47.5 ±15.3 years | IA:1 ;IB:15 IIB:3; IIIB:5 | SCNEC :18 LCNEC:6 | IHC(NA) | Intensity > ‘‘1+" | 8(33.3)% | <235.7 months | 6 | OS | E |

**SCC**: squamous cell carcinoma; **AC**: adenocarcinoma; **ASCC**, adenosquamous cell carcinoma; **SCNEC**: small cell neuroendocrine carcinoma; **LCNEC**: large cell neuroendocrine carcinoma; **EGFR**, epidermal growth factor receptor; **NA**: not available; **OS**: overall survival; **DFS**: disease-free survival; Treatment describes whether the patients received surgery (**S**), radiotherapy (**RT**), chemotherapy (**CHT**),chemoradiation (**CRT**), neoadjuvant chemotherapy (**nCHT**). For predominant treatment, in parenthesis, ‘‘**S’**’ means the patients were mainly treated by surgery; ‘‘**CR**’’ means the

patients were predominantly treated by chemoradiation (namely, treated by RT, CHT, CRTand/or nCHT); and ‘‘**M**’’ means mixed treatment. ‘‘**NA**’’ means the message was not available. Assay (p/m) is described as polyclonal antibody (**p**) or monoclonal

antibody (**m**); **IHC**: immunohistochemistry; **EIA**: Enzyme immunoassay. study quality is listed using the results of reported

questionnaire (Supplementary Table 1). **HR**: hazard ratio, obtained by estimated (**E**) or reported in text (**R**). ‘‘**M**’’ means the HR come from multivariate analysis, and the others were come from univariate analysis.
