# Supplementary material for: Prognostic Impact of Epidermal Growth Factor Receptor Overexpression in Patients with Cervical Cancer: A Meta-Analysis
Source: PLoS One. 2016 Jul 20;11(7):e0158787. doi: 10.1371/journal.pone.0158787 (PMC4954718; doi:10.1371/journal.pone.0158787)
Supplement: S2 Table — (DOC) [file pone.0158787.s002.doc]

Supplementary Table 2: quality rating score

| **Study** | **Is the population under study defined with in- and exclusion criteria?** | **Were patient data prospectively collected?** | **Are the main prognostic patient and tumor characteristics presented?^1^** | **Is the IHC or EIA staining protocol specified?^2^** | **Were stainings evaluated by > 1 observer?** | **Is the study endpoint defined?** | **Is the time of follow up specified?** | **Is loss during analysis or follow up described?** | **Quality rating** |
| --- | --- | --- | --- | --- | --- | --- | --- | --- | --- |
| Milan Vosmik 2013 | 1 | 0 | 0 | 0 | 1 | 1 | 1 | 1 | 5 |
| Halle, C. 2011 | 1 | 1 | 0 | 1 | 0 | 1 | 1 | 1 | 6 |
| Giovanna 2011 | 1 | 0 | 1 | 1 | 1 | 1 | 1 | 1 | 7 |
| Eijsink, J. J2010 | 1 | 1 | 1 | 1 | 1 | 1 | 1 | 0 | 7 |
| Noordhuis2009 | 1 | 1 | 1 | 1 | 1 | 1 | 1 | 0 | 7 |
| Yamashita, H2008 | 1 | 0 | 1 | 1 | 0 | 1 | 1 | 1 | 6 |
| Kersemaekers, A. M1999 | 0 | 0 | 1 | 1 | 1 | 1 | 1 | 0 | 5 |
| Perez-Regadera, J.2011 | 1 | 1 | 0 | 1 | 0 | 1 | 1 | 1 | 6 |
| Fuchs, I.2007 | 0 | 0 | 0 | 1 | 0 | 1 | 1 | 0 | 3 |
| Lee, C. M2005 | 1 | 0 | 0 | 1 | 0 | 1 | 1 | 0 | 4 |
| Farley, J2011 | 1 | 0 | 0 | 1 | 0 | 1 | 0 | 0 | 3 |
| Kim, Y. T.2002 | 0 | 1 | 1 | 1 | 0 | 1 | 1 | 0 | 5 |
| Kristensen, G. B1996 | 1 | 0 | 0 | 1 | 0 | 1 | 1 | 1 | 5 |
| Scambia, G.1998 | 0 | 1 | 1 | 1 | 1 | 1 | 1 | 0 | 6 |
| Baltazar, F.2007 | 0 | 0 | 0 | 1 | 0 | 1 | 0 | 0 | 2 |
| Klida2011 | 0 | 0 | 0 | 1 | 1 | 1 | 1 | 0 | 4 |
| K. OKA, 1997a(SCC) | 1 | 0 | 1 | 1 | 0 | 0 | 1 | 1 | 5 |
| K. OKA, 1997b(ACC) | 1 | 0 | 1 | 1 | 0 | 0 | 1 | 1 | 5 |
| Nagai, N2000 | 0 | 1 | 0 | 1 | 1 | 1 | 1 | 0 | 5 |
| Cho, N. H.2003 | 1 | 0 | 1 | 1 | 0 | 1 | 1 | 0 | 5 |
| Kim, G. E.2004 | 1 | 0 | 1 | 1 | 1 | 1 | 1 | 0 | 6 |
| Bodner, K2011 | 1 | 0 | 1 | 1 | 0 | 0 | 1 | 0 | 4 |
| Tangjitgamol, S2005^47^ | 1 | 0 | 0 | 1 | 1 | 1 | 1 | 1 | 6 |

Abbreviations: IHC = immunohistochemistry; EIA= Enzyme immunoassay

^1^At least four of the following characteristics: age at diagnosis, FIGO stage, tumor type, differentiation grade and residual tumor after primary surgery.

^2^At least four of the following criteria: antigen retrieval, primary antibody, dilution, detection method, cut-off value for positive expression:
