# Supplementary material for: Prognostic Impact of Epidermal Growth Factor Receptor Overexpression in Patients with Cervical Cancer: A Meta-Analysis
Source: PLoS One. 2016 Jul 20;11(7):e0158787. doi: 10.1371/journal.pone.0158787 (PMC4954718; doi:10.1371/journal.pone.0158787)
Supplement: S3 Table — (DOC) [file pone.0158787.s003.doc]

Supplementary table 3: Overview of studies that were excluded because of insufficient data

| Study | Year of publication | Data collection | country | No. in study (of deaths/recurrence) | notes |
| --- | --- | --- | --- | --- | --- |
| Leung, T. W.2001^1^ | 2001 | retrospective | Hong Kong.China | 110(NA) | mentioned that there was not any significant difference in survival (p=0.19) between those with moderate/strong expression for EGFR and those without |
| Pfeiffer, D.1989^2^ | 1989 | retrospective | Germany | 52(2) | without sufficient data to estimate HR and 95%CI |
| Perez-Regadera, J.2009^3^ | 2009 | prospective | Spain | 170(NA) | without sufficient data to estimate HR and 95%CI |
| Ngan, H.2001^4^ | 2001 | retrospective | Hong Kong.China | 101(NA) | mentioned that EGFR bear no prognostic significance in cervical cancer |
| Skomedal, H.1999^5^ | 1999 | retrospective | Norway | 74(NA) | mentioned that EGFR was not found to have an independent prognostic value |
| Hove, M. G.1999^6^ | 1999 | retrospective | USA | 22(11) | mentioned that EGFR appears to have little prognostic value in stage Ib SCC of the uterine cervix |
| Lindstrom, A. K.2007^7^ | 2007 | retrospective | Sweden | 128(NA) | without sufficient data to estimate HR and 95%CI |

**References:**

1. Leung TW, Cheung AN, Cheng DK, Wong LC, Ngan HY. Expressions of c-erbB-2, epidermal growth factor receptor and pan-ras proto-oncogenes in adenocarcinoma of the cervix: correlation with clinical prognosis. Oncol Rep 2001;8:1159-64.

2. Pfeiffer D, Stellwag B, Pfeiffer A, Borlinghaus P, Meier W, Scheidel P. Clinical implications of the epidermal growth factor receptor in the squamous cell carcinoma of the uterine cervix. Gynecol Oncol 1989;33:146-50.

3. Perez-Regadera J, Sanchez-Munoz A, De-la-Cruz J, Ballestin C, Lora D, Garcia-Martin R, Mendiola C, Alonso L, Alba E, Lanzos E. Negative prognostic impact of the coexpression of epidermal growth factor receptor and c-erbB-2 in locally advanced cervical cancer. Oncology-Basel 2009;76:133-41.

4. Ngan H, Cheung A, Liu SS, Cheng D, Ng TY, Wong LC. Abnormal expression of epidermal growth factor receptor and c-erbB2 in squamous cell carcinoma of the cervix: Correlation with human papillomavirus and prognosis. Tumor Biol 2001;22:176-83.

5. Skomedal H, Kristensen GB, Lie AK, Holm R. Aberrant expression of the cell cycle associated proteins TP53, MDM2, p21, p27, cdk4, cyclin D1, RB, and EGFR in cervical carcinomas. Gynecol Oncol 1999;73:223-8.

6. Hove MG, Dinh TV, Hannigan EV, Lucci JR, Chopra V, Smith ER, To T. Oncogene expression and microvessel count in recurrent and nonrecurrent stage Ib squamous cell carcinoma of the cervix. J Reprod Med 1999;44:493-6.

7. Lindstrom AK, Stendahl U, Tot T, Lidstrom BM, Hellberg D. Predicting the outcome of squamous cell carcinoma of the uterine cervix using combinations of individual tumor marker expressions. Anticancer Res 2007;27:1609-15.
